# Stewardship opportunities in peripartum infections: a review of quality improvement initiatives and future directions

**DOI:** 10.1017/ash.2025.10121

**Published:** 2025-09-05

**Authors:** Pamela Bailey, Grace Pazienza, Alexia Foy-Crowder, Lance Schacht, Mattie Jo Thomas, Joseph Kohn, Sarah Withers, Jessica Britt, Sean Stuart, Amy Crockett

**Affiliations:** 1 University of South Carolina School of Medicine, Columbia, SC, USA; 2 Department of Internal Medicine, Division of Infectious Diseases, Prisma Health Midlands, Columbia, SC, USA; 3 Department of Internal Medicine, Prisma Health Midlands, Columbia, SC, USA; 4 Department of Pharmacy, Prisma Health Midlands, Columbia, SC, USA; 5 Department of Pharmacy, Prisma Health Upstate, Greenville, SC, USA; 6 Department of Obstetrics and Gynecology, Division of Maternal/Fetal Medicine, Prisma Health Upstate, Greenville, SC, USA; 7 University of South Carolina School of Medicine, Greenville, SC, USA

## Abstract

Antimicrobial resistance is an urgent public health threat, and despite significant consumption of antimicrobials in pregnancy, there remain opportunities for improvement of their use in the obstetric population. Improvement in antimicrobial utilization can be streamlined by assessing baseline characteristics, utilization of diagnostic testing, awareness of peripartum protocols, and recognition of penicillin allergies. In a single healthcare system including 8 obstetric hospitals, an administrative review identified 199 different regimens used among 8,528 patients based on American College of Obstetrician and Gynecologists (ACOG) guidelines. Other notable factors include 65.6% of patients having no cultures obtained despite being started on empiric antibiotics, duplicative coverage when multiple clinical scenarios overlap, and a high incidence of reported penicillin allergies with obstetric providers lacking comfort to reconcile and de-label allergies. By reviewing these individual aspects, this can highlight opportunities for improvement of antimicrobial use and stewardship in obstetric populations.

## Introduction

There are ongoing significant increases in antimicrobial-resistant infections in hospitalized patients in the United States, emphasizing the importance of antimicrobial stewardship.^1^ The special population of obstetric patients has not had significant focus in prior reports about antimicrobial stewardship opportunities. With the 2022 *Dobbs v. Jackson Women’s Health Organization* decision by the Supreme Court to remove federal protections for abortion, subsequent increases in maternal sepsis rates emphasize that research that is needed in this population and diseases state.^2,3^

Eighty percent of prescription medications used during pregnancy are antimicrobials, and 20–25% of pregnant persons will receive an antimicrobial.^4^ Intraamniotic infection (IAI) or chorioamnionitis is an intrapartum clinical diagnosis necessitating antimicrobials. Treatment delay for confirmatory test results can lead to maternal and fetal morbidity, therefore triggering the need for urgent empiric antimicrobial initiation.^5^ The American College of Obstetricians and Gynecologists (ACOG) defines “suspected IAI” as persistently elevated temperatures plus findings of purulent cervical discharge, maternal leukocytosis, and fetal tachycardia.^3,6^ These variable and relatively complex diagnostic criteria (ie, meeting specific thresholds for each value) may introduce clinical confusion and lead to overtreatment. This may be additionally complicated by group B *Streptococcus* (GBS) carriage, surgical site infection (SSI) prophylaxis in cesarean delivery, or antimicrobial allergies.

In reviewing current practices at a single healthcare system with eight obstetric hospitals, we have identified opportunities for improvement in antimicrobial stewardship. In undertaking quality improvement projects, three different cohorts were reviewed including cohort 1 (all peripartum infections), cohort 2 (chorioamnionitis only), and cohort 3 (a large administrative sub-analysis based on ICD-10 codes). This will be illustrated through an assessment of current practices and baseline antimicrobial utilization, current diagnostic practices, current protocols regarding protocols, and antimicrobial allergies. In reviewing the current climate for management of antimicrobial utilization in obstetric patients, we aim to highlight opportunities for improvement in antimicrobial stewardship.

## Assess baseline antimicrobial utilization

An initial step is to assess utilization of traditional therapies such as ampicillin and gentamicin, plus or minus clindamycin (ie, traditional or ‘triple therapy’) for IAI. There has been significant variation documented in practice.^7^ Within our 8-obstetric hospital healthcare system, an administrative review within the electronic medical record (EMR) of 8,528 patients receiving the most common antimicrobials per ACOG guidelines demonstrated 199 separate regimens utilized.

In our initial evaluation, cohort 1 was established utilizing ICD-10 codes for all peripartum infections (ie, chorioamnionitis, endometritis, septic abortion) from April 2016 to June 2023. A random sample of 350 patients was identified. The average age was 26.3 years (range: 19–41). 63.1% (*n* = 221) were Caucasian, and 27.4% (*n* = 96) were Black; 24.3% (*n* = 85) identified as Hispanic ethnicity. Most patients delivered vaginally (*n* = 216, 61.7%) with approximately one–third (*n* = 125, 35.7%) delivering via cesarean delivery; 9 (2.6%) had a septic abortion. There were 14 different antimicrobials given initially to these patients; 334 patients received at least two antimicrobials, with 192 patients receiving three. 13 patients (3.7%) received >5 antimicrobials while in hospital. Most patients received a short duration of antibiotics of less than 2 days, but the range extended from 1 dose to 14 days in hospital and many discharged with prescriptions for 10–14 days courses.

## Assess current diagnostic practices

Once we had established baseline data, we identified cohort 2, patients with chorioamnionitis only, as a random sample of 301 patients managed within our healthcare system from April 2016 to October 2023. Most (*n* = 281, 93.4%) met ACOG diagnostic criteria for chorioamnionitis on chart review, but 20 (6.6%) did not have documented indications for initiation of treatment. There was no statistically significant association between number of symptoms meeting diagnostic criteria and fevers (*p* = .55) between cefoxitin patients or triple therapy patients; fever is the predominant symptom emphasized in the guidelines (Table 2).

When reviewing baseline practices in all patients with suspected IAI who received traditional triple therapy (cohort 1, *n* = 350), few patients identified with these infections had culture data even sent (no cultures sent, *n* = 225, 65.6%), while 38 (11.1%) had positive cultures and 80 (23.3%) had cultures with negative results. Of the 38 with positive cultures, 6 (15.8%) had bacteremia, 27 (71.1%) had bacteriuria, with 5 (13.2%) positive placental/amniotic fluid cultures positive, and 3 (7.9%) skin/wound culture positive.

In cohort 2 (chorioamnionitis only, *n* = 301), few (*n* = 59, 19.6%) patients had cultures collected, and of these 21 (35.5%) were positive including placental (*n* = 10, 47.6%) and blood (*n* = 2, 9.5%). There was not a difference in patients with positive cultures compared to patients with negative or no cultures in fevers (*p* = .77) or number of suspected IAI signs (*p* = .11).

ACOG guidelines distinguish between confirmed and suspected IAI. Confirmed IAI generally refers to IAI that is proven by microbiology testing (eg, amniotic fluid gram stain or culture). Furthermore, cultures and histologic analysis are noted to be meaningful only in research settings and not for obstetric providers in managing a laboring patient as delay in treatment may result in unacceptable morbidity and mortality of both the laboring patient and their neonate.^3^ ACOG guidelines, therefore, suggest that antibiotic treatment should be initiated for suspected IAI (ie, fever plus an additional clinical criterion) and considered for isolated fever alone.^1^ This likely results in overtreatment, with many patients being exposed to broad-spectrum antibiotics due to nonspecific clinical criteria; this is supported by our observations in cohort 2.

## Assess antimicrobial protocols

IAI requires urgent antimicrobial administration to prevent progression to sepsis. Obstetric patients may also require GBS prophylaxis or SSI prophylaxis. Many of these patients may receive duplicative antimicrobial coverage due to multiple clinical scenarios colliding, with noted overlapping spectrum of activity.

Utilizing cohort 2 (*n* = 301), comparisons were made between the traditional triple therapy regimen prior to June 2023 and the switchover to cefoxitin after June. 19 patients (6.3%) received antimicrobials for all three indications of IAI, GBS prophylaxis, and SSI prophylaxis. Rates of SSI prophylaxis were appropriately high for patients with cesarean delivery (*n* = 105/113) and GBS carriage (*n* = 43/49). While appropriateness for indication was generally excellent, we saw changes in prescribing patterns after June 2023 in double *β*-lactams. There was a significant difference in patients receiving two cephalosporins in the traditional (*n* = 1, 2.8%) and revised (*n* = 34, 97.1%) groups (*p* < .001), and in those receiving two types of penicillin coverage (traditional, *n* = 26, 86.7%) vs revised (*n* = 4, 14.3%) (*p* = .008). This is presumably related to cefazolin/cefoxitin utilization for SSI and chorioamnionitis, substituting in for ampicillin (IAI) and penicillin (GBS) in the triple therapy regimens, though the fact of duplicative beta-lactam coverage remained. After cefoxitin-based guidelines implementation in our health system (June 2023), there was a significant reduction in the number of antimicrobials to which patients were exposed in cohort 2 (*p* < .01, Table 1). Notably, maternal clinical outcomes were non-inferior with utilization of monotherapy with cefoxitin compared to the traditional triple therapy.^8^

**Table 1.** Exposure to antimicrobials in cohort 2

Number of antimicrobials	Traditional triple therapy (precefoxitin Implementation) (*n* = 195)	Revised (postcefoxitin implementation) (*n* = 106)
1	195 (100%)	106 (100%)
2	193 (99%)	57 (54%)
3	108 (55%)	42 (40%)
≥4	111 (57%)	16 (15%)

Cohort 3, an administrative subanalysis review of 8,528 patients based on ICD-10 diagnoses for IAI, sorted by antimicrobial regimens identified 199 separate regimens prescribed, 92 (46.2%) of which had duplicative spectrum of activity. This represented 551 (6.5%) individual patients; the top three regimens were cefazolin and penicillin (*n* = 126), cefazolin and ampicillin (*n* = 51), and cefazolin and cefoxitin (*n* = 47). 33 (16.6%) were in line with ACOG guidelines. 12 of these 33 (36.4%) were ACOG-endorsed regimens with duplicative spectrum of activity.

## Assess antimicrobial allergies

In cohort 1 (triple therapy, *n* = 350), 83.1% of patients had no antimicrobial allergies, while 11.4% reported a penicillin allergy. In cohort 3 (administrative review, *n* = 8,528) notably, 3,957 (46%) patients had penicillin allergies listed in the chart and 816 (9.5%) patients had cephalosporin allergies. Despite multiple touchpoints with obstetric providers during the course of pregnancy, allergies remained in place. In reviewing guidelines with OBGYN providers, they indicated significant concern about penicillin allergies in their patients and an acknowledgement of the inaccuracies of the allergy profile in the EMR. However, they felt unable to perform accurate allergy history reconciliation and expressed low comfort level to de-label the penicillin allergies.

## Summary of stewardship opportunities

As noted here, there are significant opportunities for quality improvement initiatives that should be undertaken in obstetric patients to improve antimicrobial prescribing. There is significant variability in prescribing, compounded by the various protocols for GBS, SSI, or IAI as well as allergy profiles.^7^ Clinical decision support tools in the EMR may be beneficial; we went to cefoxitin as first-line therapy, adjusted order sets accordingly, and saw improvement in reduction of antimicrobial exposures in patients. However, this introduced regimens with duplicative spectrum of activity, particularly around administration of both cefazolin and cefoxitin, which indicates there is still room for improvement, particularly where IAI management overlaps with GBS and SSI prophylaxis.

Updating patient allergies will lead to decreased variability in antimicrobial prescribing in this population. Notably, penicillin allergy de-labelling is a critical opportunity not just for peripartum infections but also for treatment of syphilis and decreasing congenital syphilis, a current scourge.^9^ Frequent visits during the course of routine care provide excellent touchpoints where clarification about a patient’s medication allergies could be performed, with appropriate next steps recommended. Penicillin allergy de-labeling in pregnant patients with history of low-risk allergy symptoms and penicillin skin testing are safe allergy assessment strategies in pregnant patients; allergy evaluation increases the number of patients receiving first-line antimicrobial prophylaxis for both SSI and GBS.^10^

There is also significant clinical variability in assessing maternal sepsis risk, with few tests done at the bedside that can help inform empiric antimicrobial prescribing, yet this must be weighed against the threat of antimicrobial resistance and limited microbiology studies. Notable advances in technology have dramatically changed the understanding of the flora and microbiome of the vagina and placenta; however, the pathogenicity of some of the organisms identified is uncertain and more studies are needed.^11^ Multiple studies from the 1970s and 1980s acknowledge that the spectrum of activity of current ACOG-endorsed antimicrobial regimens miss organisms such as *Enterococcus* spp. and *Ureaplasma*s, yet have good clinical outcomes, but we have better tests in the modern era and need to apply them in this arena.^12^

Additionally, current clinical guidance for obstetric providers suggests that cultures in the setting of IAI are not meaningful for most clinicians in determining empiric therapy, and only offer impact in research settings “until better and less invasive intrapartum diagnostic tools become available” despite advancements in rapid microbiological diagnostics.^3^ This can limit opportunities for targeted therapy towards dominant pathogenic organisms or confirmed diagnoses of infectious syndromes.

Limitations of our conclusions include sampling bias and generalizability; as these observations arose from quality improvement initiatives centered on reviewing practice within our system, they may not be applicable to other systems or useful for power calculation for larger prospective studies. Additionally, errors may have been made in chart abstraction or in coding; for example, the penicillin allergy coding is a discrete variable and trends higher than expected in the administrative data cohort 3.

While obstetric patients utilize a significant amount of antimicrobials, they have not traditionally been included in many stewardship initiatives despite the significant opportunities that exist, and future projects should include this patient population to improve delivery of quality care to this vulnerable population.

**Table 2.** Diagnostic criteria for IAI including categories of maternal fever and clinical findings in cohort 2

	Fever >39°C (*n* = 42)	Fever 38.0–38.9 >30 minutes (*n* = 144)	Fever 38.0–38.9 <30 minutes or no maternal fever (*n* = 120)
Suspected IAI:			
Maternal tachycardia	28 (66.7)	107 (74.3)	67 (55.8)*
Maternal leukocytosis	24 (57.1)	60 (41.7)	46 (38.3)*
Fetal tachycardia	19 (45.2)	56 (38.9)	52 (43.3*
Fundal tenderness	7 (16.7)	12 (8.3)	13 (10.8)*
Purulent discharge	3 (7.1)	4 (2.8)	15 (12.5)*
No clinical findings	3 (7.1)	17 (11.8)*	20 (16.7)*
Confirmed IAI:			
Positive amniotic fluid gram stain	0	0	0
Positive amniotic fluid glucose test	0	0	0
Positive amniotic fluid culture	0	0	0
Placental pathology consistent with infection or inflammation	20 (48.8)	52 (36.1)	45 (38.5)
None of these documented	21 (51.2)	92 (63.9)	72 (61.5)

* Indicates documentation for these patients is not compliant with ACOG diagnostic criteria for IAI. Note: Patients may be in multiple categories of fever.

## Data Availability

Data Available upon reasonable request to communicating author.
